# Surgical Management of a Maxillary Odontogenic Keratocyst: A Clinical Case Report

**DOI:** 10.3390/dj13110514

**Published:** 2025-11-05

**Authors:** Ioan Sirbu, Ionut Cosmin Nisipasu, Pasquale Savino, Andreea Mihaela Custura, Elisei Adelin Radu, Vladimir Nastasie, Valentin Daniel Sirbu

**Affiliations:** 1 Discipline of Implanto-Prosthesis Therapy, Faculty of Dentistry, “Carol Davila” University of Medicine and Pharmacy, 37 Dionisie Lupu Street, District 2, 020021 Bucharest, Romania; 2 Private Practice, Via San Marco, Via II Vinella, II, 8, 81028 Santa Maria A Vico, CE, Italy; 3 Discipline of Dental Prostheses Technology, Faculty of Dentistry, “Carol Davila” University of Medicine and Pharmacy, 37 Dionisie Lupu Street, District 2, 020021 Bucharest, Romania; 4 Doctoral School, Faculty of Dentistry, “Carol Davila” University of Medicine and Pharmacy, 050474 Bucharest, Romania

**Keywords:** odontogenic keratocyst, maxillary sinus, PRF, case report

## Abstract

**Introduction**: Odontogenic keratocyst (OKC) is a locally aggressive cystic lesion derived from remnants of the dental lamina. It is most commonly located in the posterior mandible, while maxillary involvement is rare and poses diagnostic and surgical challenges due to its proximity to critical anatomical structures. This case report describes the surgical management of a maxillary OKC with an uncommon localisation. **Methods**: A 50-year-old male presented with an asymptomatic swelling in the posterior maxilla. Cone beam computed tomography (CBCT) revealed a well-defined unilocular radiolucency extending toward the maxillary sinus floor. Surgical management included complete enucleation and peripheral curettage, followed by histopathological confirmation. The defect was left to heal naturally through bone regeneration without the need for grafting. **Results**: Intraoperatively, a thin pearly white cystic capsule and buccal cortical thinning were observed, consistent with OKC. The lesion was enucleated intact, without rupture or sinus perforation. Histology confirmed the diagnosis. Postoperative healing was uneventful, with radiographic follow-up at one month showing favourable healing changes. **Conclusions**: Careful surgical planning combined with advanced imaging facilitates safe and effective management of OKCs in uncommon maxillary sites. Enucleation with peripheral curettage provided satisfactory short-term outcomes. Long-term follow-up remains essential due to the risk of recurrence.

## 1. Introduction

Odontogenic keratocyst (OKC) is a rare, non-cancerous cystic lesion that forms from the remains of the dental lamina and is aggressive in the area where it develops. It constitutes up to 5–15% of all jaw cysts and is distinguished by its high recurrence rates and the potential for substantial bone destruction [1]. Recent epidemiological studies further confirm the variability in incidence and recurrence rates depending on population and geographic setting, such as a six-year retrospective study in South Sulawesi, Indonesia [2].

Specifically affecting the premolar, molar region, and ramus of the mandible, OKC is more frequently found in the mandible than the maxilla. The maxillary sinus is rarely affected by KCOT, with only a limited number of cases documented in the literature [3].

Philipsen originally wrote about odontogenic keratocyst (OKC) in 1956 [4]. Since then, it has been known for its unique histological characteristics and aggressive biological behaviour. The World Health Organisation (WHO) changed the name of the lesion to keratocystic odontogenic tumour (KCOT) in 2005 since it grew like a tumour, came back often, and had genetic links such as PTCH1 mutations. But in the most recent WHO editions, the classification has gone back to a cystic entity, and many writers still use the old term odontogenic keratocyst (OKC) in academic as well as clinical contexts. The primary reason for this is the necessity for terminological clarity in multidisciplinary communication, surgical manageability, and the non-neoplastic histology [5]. In addition, molecular insights are expanding, with recent bioinformatics analyses implicating oxidative stress-related pathways and hub genes in OKC pathogenesis [6].

OKC is frequently asymptomatic and may develop to a substantial size before clinical detection, despite its histologically distinctive features, which include a parakeratinized stratified squamous epithelium with a palisaded basal layer [7]. Radiographically, it may resemble other cystic lesions, such as dentigerous cysts, particularly when they are associated with unerupted teeth [8,9].

Without pathognomonic symptoms, these lesions are typically found by chance during routine imaging. The presence of this overlap in radiographic appearance requires a comprehensive radiological evaluation, preferably through cone beam computed tomography (CBCT), to assess the proximity of vital structures, cortical involvement, and lesion boundaries. However, a clear diagnosis needs histological confirmation because OKC is individual since it has a parakeratinized stratified squamous epithelium, a palisaded basal layer, and no inflammation in cases that are not infected. Only this combination of imaging and microscopic testing can guarantee an accurate diagnosis and the right treatment strategy [10].

Several therapeutic approaches have been explored, ranging from conservative decompression and marsupialisation to surgical excision. Complete enucleation with peripheral curettage is a widely accepted method for well-delimited lesions, as it effectively balances tissue preservation with therapeutic efficacy [11].

Because the OKC presence in the maxillary sinus is uncommon, the radiographic image may be indistinguishable and misunderstood. There is debate regarding the origin of OKC in the maxillary sinus or its involvement. The entrapment of odontogenic epithelium within the sinus is likely the result of the close anatomic relationship between the dental lamina and developing antrum or the primordium of the canine and the sinus floor, as well as the close relationship between the maxillary molars and antrum floor, which leads to its involvement [12].

The clinical case of a large maxillary OKC, a less common localisation, is presented in this article. The case was effectively managed through complete enucleation and peripheral curettage. The intraoperative photographic documentation improves comprehension of the surgical technique and demonstrates the favourable postoperative outcome.

## 2. Materials and Methods

**Figure 1:**
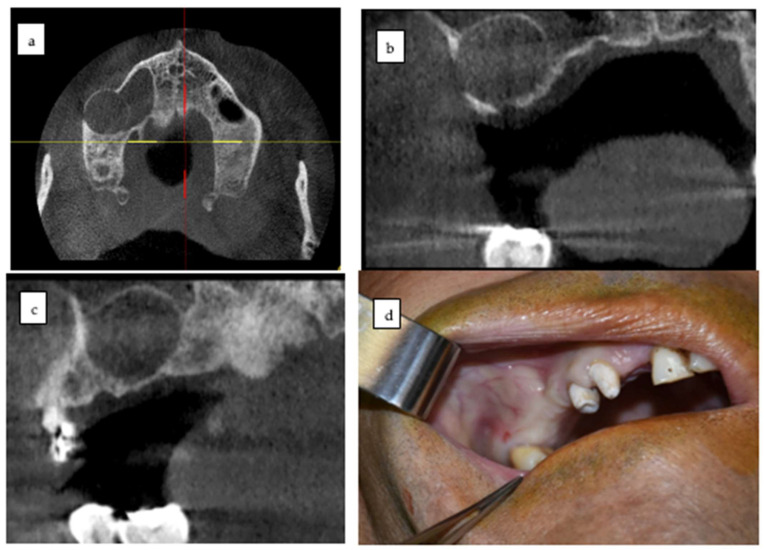
Preoperative CBCT views of the maxillary odontogenic cyst in quadrant I. (**a**) Axial section showing the well-defined hypodense lesion with cortical thinning. (**b**) Coronal section illustrating bucco-palatal expansion and the relationship of the cyst with adjacent teeth and maxillary sinus floor. (**c**) Sagittal section demonstrating the anteroposterior extension of the lesion. (**d**) Preoperative intraoral photograph showing vestibular swelling and mucosal distension in the posterior left maxilla, corresponding to the underlying cystic lesion.

A 50-year-old male patient presented with a progressive, asymptomatic swelling in the posterior region of the left maxilla. In the absence of signs of acute infection, the clinical examination revealed an asymmetrical expansion with variable consistency. A well-defined, unilocular radiolucent lesion extending approximately 19.0 mm mesiodistally and 16.0 mm vertically, from the alveolar crest toward the floor of the maxillary sinus, in the alveolar regions of teeth 1.6 and 1.7, was identified using cone beam computed tomography (CBCT) (Figure 1). The presumptive diagnosis was an odontogenic cyst, which was subsequently confirmed by histopathological analysis as an odontogenic keratocyst (OKC). The surgical treatment was designed to include complete enucleation of the lesion, peripheral curettage, and long-term postoperative follow-up.

### 2.1. Anaesthesia and Incision

The procedure was conducted under local anaesthesia, utilising an infraorbital and posterior superior alveolar nerve block. The lesion was optimally accessed by making a linear vestibular incision from the region of tooth 1.5 to the distal aspect of 1.8. To conserve keratinised tissue and facilitate flap repositioning, the incision was positioned 3–4 mm above the mucogingival junction.

### 2.2. Flap Design and Elevation

From the mesial aspect of 1.5 to the posterior tuberosity area, a full thickness mucoperiosteal flap was elevated. To prevent unnecessary trauma to the periosteum and to thoroughly expose the cortical expansion caused by the lesion, flap elevation was performed with careful attention. This method guaranteed comprehensive access to the cystic formation and enabled a sufficient view of the surgical field.

### 2.3. Identification and Enucleation of the Lesion

**Figure 2:**
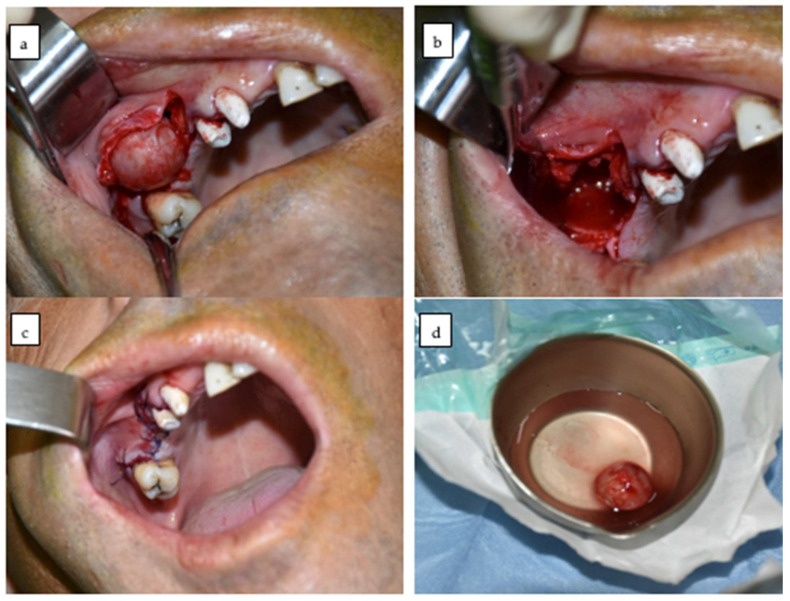
Intraoperative and postoperative views. (**a**) Exposure and dissection of the cystic capsule through a vestibular approach, showing the thin, pearly white membrane characteristic of an odontogenic keratocyst. (**b**) Post-enucleation intraoral view of the surgical site. (**c**) Primary closure achieved with interrupted sutures. (**d**) Excised specimen, extraoral view.

Buccal cortical thinning and protrusion were observed upon flap reflection. Sharp curettes were employed to meticulously identify and dissect the cystic capsule from the encircling bone. Special precautions were taken to preserve the integrity of the cystic wall and prevent rupture during the single-piece enucleation. The lesion exhibited the typical pearly white, thin-walled aspect of odontogenic keratocysts (Figure 2a) [13].

### 2.4. Peripheral Curettage

Sharp surgical curettes were employed to perform peripheral curettage of the internal bony cavity following enucleation. This step was crucial for the removal of residual epithelial remnants and for the reduction in the risk of recurrence, particularly in the regions where the capsule had a strong adherence to the bone. The procedure did not reveal any communication with the maxillary sinus.

### 2.5. Cystic Cavity Management

Following the completion of enucleation and curettage, the intraosseous cavity was assessed (Figure 2b). No perforation into the maxillary sinus was observed in the bony walls. No grafting material or membrane was employed due to the favourable morphology and absence of oroantral communication. The cavity was allowed to heal naturally through secondary bone regeneration.

### 2.6. Suture and Postoperative Management

The surgical site was irrigated with sterile saline solution, and haemostasis was checked. Layered closure was used using interrupted sutures for the mucosa (Figure 2c) and 4–0 resorbable sutures for the periosteum. After receiving postoperative instructions and a prescription for antibiotics and analgesics, the patient was discharged on the same day. Follow-up consultations were scheduled to evaluate any indications of an early recurrence and monitor healing.

### 2.7. Medication Administered Following Surgery

The patient was prescribed a 7-day course of amoxicillin-clavulanic acid (875 mg/125 mg, twice daily), ibuprofen 400 mg as needed for pain, and chlorhexidine 0.12% mouthwash twice daily for 10 days.

### 2.8. Histopathological Processing

The removed cystic membrane was immediately immersed in 10% buffered formalin [14,15] (Figure 2d) and sent for histological assessment. Standard haematoxylin and eosin (H&E) staining was conducted to verify the diagnosis and exclude neoplastic transformation [16]. The macroscopic evaluation revealed a cystic formation with a maximum diameter of 1.5 cm. The diagnosis of odontogenic keratocyst was confirmed by microscopically examining the cystic lining, which consisted of a parakeratinized stratified squamous epithelium of 6–8 cell layers with a palisaded basal layer of hyperchromatic nuclei and a corrugated parakeratin surface [17].

### 2.9. Follow-Up Protocol

**Figure 3:**
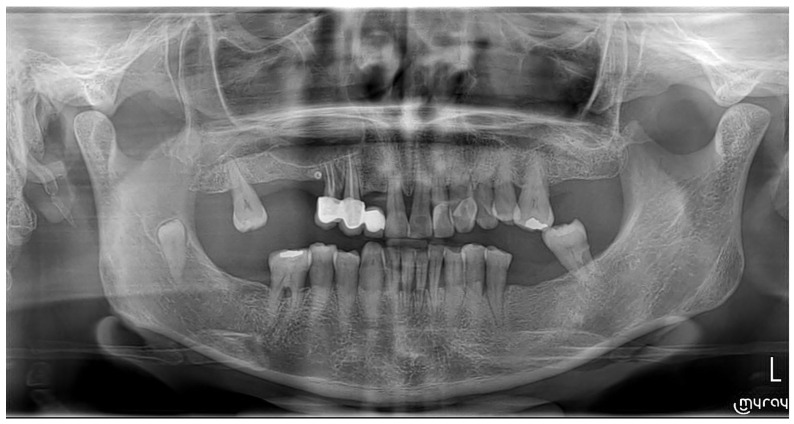
Two-year follow-up panoramic radiograph demonstrating stable bone healing in the region of the previously enucleated odontogenic cyst, with no evidence of recurrence.

The patient was monitored postoperatively at 7, 14 days, and 1 month. The healing process was uneventful, with no indications of infection, dehiscence, or paraesthesia. Radiographic follow-up at one month suggested early healing changes, although true new bone formation cannot be confirmed radiographically at this stage; bone consolidation is generally appreciable radiographically after 6 weeks or more [18,19]. Long-term follow-up was also carried out, and a panoramic radiograph taken at 2 years postoperatively confirmed complete bone healing in the operated region, with no evidence of recurrence (Figure 3). In the postoperative panoramic radiograph (Figure 3), a radiopaque image was noted distal to tooth #14. While the exact nature of this image remains uncertain, it is most likely related to a metallic artefact. However, no clinical relevance or pathological association was detected during patient evaluation. Regarding the radiolucent area observed in the right mandibular ramus adjacent to the impacted third molar, additional assessment with CBCT is planned for the near future to provide further clarification. At the time of manuscript submission, this investigation had not yet been performed.

## 3. Discussion

Odontogenic keratocysts are recognised by their aggressive nature, high recurrence rate, and histopathological uniqueness [20]. While OKC is most frequently detected in the mandible, particularly the posterior body and ramus, it is uncommon to find OKC in the maxillary sinus, which accounts for a small percentage of documented cases [21]. The case described in this article is a perfect illustration of an uncommon localisation, which presents in the posterior maxilla with sinus floor involvement. Consequently, it presents both diagnostic and surgical challenges.

Until they reach considerable size, maxillary OKCs may not exhibit any clinical symptoms, and they frequently resemble other benign cystic lesions on radiographs [17]. In this instance, the lesion was identified as a result of oedema and asymmetry, and CBCT imaging was instrumental in determining the lesion’s boundaries and its proximity to the maxillary sinus. The risk of sinus perforation and the need to assess cortical bone involvement necessitated a precise radiological assessment for preoperative planning.

The unique localisation within the maxilla prompts several questions. Initially, the risk of inadvertent sinus communication during surgical excision is elevated due to the proximity to the maxillary sinus. Nevertheless, the absence of perforation in the current instance is likely attributable to the meticulous surgical technique and the lesion’s restricted vertical extent. Secondly, the hypothesis that ectopic dental epithelium may contribute to the development of OKCs in this region is substantiated by the close anatomical relationship between the dental lamina and the maxillary antrum. Third, the absence of oroantral communication enabled a more conservative postsurgical approach, which did not necessitate the use of barrier membranes or grafts.

Various treatment strategies have been suggested for the management of OKCs, each with a recurrence rate that varies. For large or high-risk lesions, conservative procedures, such as marsupialisation and decompression, are reserved, particularly in younger patients or those with syndromic conditions [22]. Although these methods preserve bone and reduce surgical morbidity, they frequently necessitate extended follow-up and have higher recurrence rates if not followed by definitive enucleation [23].

The most frequently employed and effective method for non-syndromic OKCs in accessible locations is complete enucleation followed by peripheral curettage, as demonstrated in this instance. The objective of peripheral curettage is to eradicate residual epithelial remnants that may not be visible macroscopically, thereby reducing the likelihood of recurrence. Large multicenter data also support this approach, showing lower recurrence rates with enucleation plus curettage compared to more conservative methods such as decompression or marsupialisation [24]. The adjunctive use of Carnoy’s solution, cryotherapy, or liquid nitrogen has been recommended in certain studies to further reduce recurrence rates. However, these techniques are associated with increased complication risks, including delayed healing and neurovascular damage, and were therefore not deemed appropriate for this anatomically sensitive location [25].

OKC recurrence rates vary greatly depending on the treatment approach, with enucleation alone resulting in rates ranging from 13% to 58%, and enucleation combined with curettage exhibiting improved long-term outcomes [26]. In the present situation, the surgical technique was influenced by the lesion’s well-defined nature and the patient’s overall health. The classic features of OKC—parakeratinized stratified squamous epithelium with palisaded basal cells—were confirmed by histopathological confirmation using haematoxylin and eosin staining, thereby excluding malignant transformation and confirming the diagnosis.

There were no indications of neurosensory complications or infection during the postoperative period. Rigorous long-term surveillance is still very important because OKC is known to regenerate back, often years after the first surgery. This is consistent with recent long-term follow-up studies documenting recurrences even decades after initial treatment, reinforcing the necessity of extended monitoring [27].

In summary, this case emphasises the significance of a comprehensive diagnostic workup, particularly in atypical presentations like maxillary OKCs. The chosen method, which involved complete enucleation and peripheral curettage, yielded favourable results while minimising surgical morbidity. Early diagnosis, precise imaging, and meticulous surgical execution continue to be essential for achieving optimal patient outcomes and reducing recurrence.

## 4. Conclusions

This case highlights the successful management of a rare maxillary odontogenic keratocyst located near the sinus floor. Careful preoperative CBCT evaluation and complete enucleation with peripheral curettage allowed for safe removal with minimal morbidity and favourable healing. The report underlines the importance of advanced imaging and meticulous surgical planning in atypical localisations. Although long-term follow-up remains essential due to the recurrence potential of OKCs, the present findings reinforce the effectiveness of this conservative surgical approach in improving patient outcomes.

## Data Availability

The data presented in this study are available on request from the corresponding author. The data are not publicly available due to privacy and ethical restrictions related to patient confidentiality.

## Abbreviations

The following abbreviations are used in this manuscript:

OKC	Odontogenic keratocyst
WHO	World Health Organisation
KCOT	Keratocystic odontogenic tumour
CBCT	Cone Beam Computed Tomography
